# The Hypothetical Protein ‘All4779’, and Not the Annotated ‘Alr0088’ and ‘Alr7579’ Proteins, Is the Major Typical Single-Stranded DNA Binding Protein of the Cyanobacterium, *Anabaena* sp. PCC7120

**DOI:** 10.1371/journal.pone.0093592

**Published:** 2014-04-04

**Authors:** Anurag Kirti, Hema Rajaram, Shree Kumar Apte

**Affiliations:** Molecular Biology Division, Bhabha Atomic Research Centre, Trombay, Mumbai, India; Saint Louis University, United States of America

## Abstract

Single-stranded DNA binding (SSB) proteins are essential for all DNA-dependent cellular processes. Typical SSB proteins have an N-terminal Oligonucleotide-Binding (OB) fold, a Proline/Glycine rich region, followed by a C-terminal acidic tail. In the genome of the heterocystous nitrogen-fixing cyanobacterium, *Anabaena* sp. strain PCC7120, *alr0088* and *alr7579* are annotated as coding for SSB, but are truncated and have only the OB-fold. *In silico* analysis of whole genome of *Anabaena* sp. strain PCC7120 revealed the presence of another ORF ‘*all4779’,* annotated as a hypothetical protein, but having an N-terminal OB-fold, a P/G-rich region and a C-terminal acidic tail. Biochemical characterisation of all three purified recombinant proteins revealed that they exist either as monomer or dimer and bind ssDNA, but differently. The All4779 bound ssDNA in two binding modes i.e. (All4779)_35_ and (All4779)_66_ depending on salt concentration and with a binding affinity similar to that of *Escherichia coli* SSB. On the other hand, Alr0088 bound in a single binding mode of 50-mer and Alr7579 only to large stretches of ssDNA, suggesting that All4779, in all likelihood, is the major typical bacterial SSB in *Anabaena*. Overexpression of All4779 in *Anabaena* sp. strain PCC7120 led to enhancement of tolerance to DNA-damaging stresses, such as γ-rays, UV-irradiation, desiccation and mitomycinC exposure. The tolerance appears to be a consequence of reduced DNA damage or efficient DNA repair due to increased availability of All4779. The ORF *all4779* is proposed to be re-annotated as *Anabaena ssb* gene.

## Introduction

Single-stranded DNA-binding proteins (SSB) are ubiquitous proteins found in all bacteria. The SSB proteins are characterised by their non-specific binding to single-stranded DNA (ssDNA) and active participation in the maintenance of genome integrity (DNA repair) as well as genetic information transfer (replication and transcription) [1]. A typical SSB monomer consists of (a) an N-terminal region having several conserved residues responsible for binding to ssDNA, tetramerisation and stabilization of monomer fold [2], and (b) a C-terminal region which displays a low sequence conservation except for the last few amino acids (known as acidic tail), and is responsible for protein-protein interactions and recruitment of DNA interactive proteins [3]. The highly conserved OB-fold has been extensively described for *Escherichia coli* SSB protein [1]. Deletion of C-terminus diminishes recruitment of other DNA interacting proteins [3], but enhances the affinity of N-terminus to ssDNA [4]. The spacer region between the N-terminal OB-fold and C-terminal acidic tail is rich in proline/glycine (P/G) residues and is thought to modulate the strength of DNA binding, possibly by distancing the highly negatively charged C-terminal end from the positively charged DNA binding N-terminal domain [5].

Nitrogen-fixing cyanobacteria, such as strains of *Anabaena* and *Nostoc* exhibit tolerance to a variety of abiotic stresses such as salinity, desiccation, heat and radiation [6], [7], strongly indicative of a robust mechanism of DNA repair in these microbes [8]. Unfortunately, genes/proteins involved in DNA metabolism of cyanobacteria have not received adequate attention. The SSB protein is a key protein involved in all DNA related cellular activities. In the genomic database of *Anabaena* sp. strain PCC7120 (hereafter referred to as *Anabaena* 7120), two ORFs ‘*alr0088’* and ‘*alr7579’* are annotated as coding for SSB-like proteins (http://genome.microbedb.jp/cyanobase/Anabaena) and exhibit 28–30% homology at amino acid level with EcoSSB, and about 42% homologous to each other. However, BLAST search [9] of the amino acid sequence of these two proteins show that the protein sequence is terminated immediately after the N-terminal OB-fold region and have no region corresponding to either the P/G spacer or the C-terminal acidic tail. Since, C-terminal acidic tail is essential for interaction with other DNA replication/repair/recombination proteins [3], it seems unlikely that the proteins encoded by these two ORFs can perform all the functions of SSB proteins. However, this does not rule out that they are genuine SSBs, since the second SSB (SsbB) of naturally transformable bacteria, such as *Bacillus subtilis*, lacks the C-terminal acidic tail, but functions as a SSB and is involved in genetic recombination [10], [11]. Alr0088 and Alr7579 exhibit about 36–38% overall homology with BsSsbB. An *in silico* search for a SSB-like protein with the C-terminal region (i.e. the spacer region and acidic tail) in the genome of *Anabaena* 7120, revealed the ORF ‘*all4779’.* The ORF encodes a 182 amino acid long protein with an N-terminal OB-fold, a P/G rich spacer region and a C-terminal acidic tail but has been annotated as a hypothetical protein possibly due to its lower homology (15–18%) with other bacterial SSBs. The homologs of the two truncated SSB-like proteins as well as hypothetical SSB-like protein of *Anabaena* 7120 are found across all cyanobacterial genomes (http://genome.microbedb.jp/cyanobase).

In the present work, we cloned, overexpressed, purified and biochemically characterised Alr0088, Alr7579 and All4779 proteins. All three proteins existed in monomeric and dimeric forms and showed differential binding to ssDNA. All4779 protein showed typical structural features, oligomerisation, ssDNA binding modes compared to *E. coli* SSB and conferred tolerance to DNA damage, upon overexpression in *Anabaena*, that identifies it as the major SSB of *Anabaena*.

## Materials and Methods

### Organism and Growth Conditions

*E. coli* cells were grown in Luria–Bertani (LB) medium at 37°C with shaking (150 rpm). When required antibiotics [34 μg chloramphenicol mL^−1^(Cm_34_), 50 μg kanamycin mL^−1^ (Kan_50_), or 100 μg carbenicillin mL^−1^ (Cb_100_)] were used in culture media. Axenic cultures of *Anabaena* 7120 were grown in BG-11 liquid medium, pH 7.0 [12] in the absence of combined nitrogen (BG-11, N^−^) under stationary conditions with continuous illumination (30 μE m^−2^ s^−1^) at 27°C±2°C. Recombinant *Anabaena* strains were grown in the presence of 10 μg neomycin mL^−1^ (Nm_10_) in BG-11 liquid media or with 25 μg neomycin mL^−1^ (Nm_25_) on BG-11 agar plates. Growth was assessed in terms of chlorophyll *a* content per ml of culture as described earlier [13]. Cell survival was assessed in terms of colony forming units (cfu) by plating 100 μl of the culture on to BG-11, N^−^ agar plates followed by incubation under illumination for 10 days as described earlier [14].

Three-day-old nitrogen-fixing *Anabaena* cultures were concentrated to a chlorophyll *a* density of 10 μg mL^−1^, prior to subjecting them to one of the following stresses: (i) 6 kGy of ^60^ Co γ-rays at a dose rate of 4.5 kGy h^−1^, or (ii) 6 days of desiccation or in humid chamber (control), or (iii) 0–4 μg mitomycinC (mitC) mL^−1^ for 30 min, or (iv) exposure to 0–1.5 kJ UV-B (280 nm) (dose rate 5 J m^−2^ sec^−1^) for different duration. Survival in response to stress, and post-stress recovery were compared with unstressed/control cultures grown under illumination at 27°C±2°C as described earlier [14].

### Generation of Plasmid Constructs for Overexpression of Proteins in *E. coli*

Different amplicons (*alr0088*, *alr7579*, *all4779*) were generated by PCR amplification of *Anabaena* 7120 genomic DNA (100 ng) using gene specific primers (as indicated in Table 1), 1 μM dNTPs and 1U Taq DNA polymerase in Taq buffer (Bangalore Genei, India). These amplicons were individually digested with *Nde*I and *Bam*HI restriction endonucleases and ligated to the expression vector pET16b (Table 2), having His_10_-tag at the 5′ end, at identical restriction sites. The resulting plasmid constructs were designated as pET*alr0088*, pET*alr7579* and pET*all4779* respectively (Table 2). DNA insert of all three plasmids were sequenced on both strands using Sanger’s dideoxy method and were found to be completely identical to the corresponding nucleotide sequences available in the genomic database. The nucleotide sequences corresponding to *alr0088*, *alr7579* and *all4779* respectively were submitted to GenBank (GenBank Accession Nos. GU225949, GU225950, GU225951).

**Table 1:** Primers used and PCR amplicons generated in this study.

Primers	Nucleotide Sequence*	R.E.	Amplicon#
*alr0088*Fwd	5′ GGCCATATGAGCATTAACATTGTC 3′	*Nde*I	*alr0088* ORF (0.35 kb)
*alr0088* Rev	5′ GGCGGATCCTTAAAAATTTTCTGGTGC 3′	*Bam*HI	
*alr7579*Fwd	5′ GGCCATATGAACTATATCAACAAA 3′	*Nde*I	*alr7579* ORF (0.38 kb)
*alr7579* Rev	5′ GGCGGATCCCTAGAAATTTGCGTTAGC 3′	*Bam*HI	
*all4779*Fwd	5′ GGCCATATGAACAGCTGTGTTTTA 3′	*Nde*I	*all4779* ORF (0.55 kb)
*all4779* Rev	5′ GGCGGATCCTAAAATGGAATATCGTC 3′	*Bam*HI	

*The restriction endonuclease (R.E.) site included in each primer is underlined and the corresponding R.E. indicated in the adjacent column.

# The amplicons generated with a given set of PCR primers are specified.

**Table 2:** Plasmids used in this study.

Plasmids	Description	Source/Reference
pET16b	Cb^r^, expression vector	Novagen
pAM1956	Kan^r^, promoterless vector with *gfpmut2* reporter gene	[19]
pBluescript (pBS)	Cb^r^, cloning vector	Lab Collection
pFPN	Cb^r^, Kan^r^, integrative expression vector	[18]
pET*alr0088*	Cb^r^, 0.35 kb *alr0088* gene cloned in pET16b at *Nde*I/*Bam*HI restriction sites	This study
pET*alr7579*	Cb^r^, 0.38 kb *alr7579* gene cloned in pET16b at *Nde*I/*Bam*HI restriction sites	This study
pET*all4779*	Cb^r^, 0.55 kb *all4779* gene cloned in pET16b at *Nde*I/*Bam*HI restriction sites	This study
pFPN*all4779*	Cb^r^, Kan^r^, 0.55 kb *all4779* gene cloned in pFPN at *Nde*I/*Bam*HI restriction sites	This study
pAM*all4779*	Kan^r^, 0.81 kb *Xma*I-*Sal*I fragment from pFPN*all4779* gene cloned in pAM1956	This study

### Overexpression and Purification of His-tagged Proteins

The plasmids pET*alr0088*, pET*alr7579* and pET*all4779* (Table 2) were transformed into *E. coli* BL21(pLysS) and transformants selected on LBCm_34_Cb_100_ plates (Table 3). Proteins were overexpressed from the respective logarithmic phase cultures of *E. coli* upon addition of 1 mM IPTG for 3 h at 37°C. The recombinant His-tagged proteins (Alr0088, Alr7579 and All4779) were extracted in lysis buffer (20 mM Tris-HCl, pH 8, 0.5 M NaCl, 5 mM imidazole and 0.1% TritonX-100) by sonication and purified by Ni-NTA affinity chromatography (Qiagen, Germany) using different concentrations of imidazole ranging from 10–1000 mM as described earlier. The proteins were eluted individually in 1 M imidazole fraction and visualised by electrophoretic separation on 14% SDS-polyacrylamide gel followed by staining with Coomassie Brilliant Blue (CBB) G-250. The proteins were quantified spectrophotometrically by measuring absorbance at 280 nm using 19480 M^−1^ cm^−1^, 20970 M^−1^ cm^−1^ and 12950 M^−1^ cm^−1^ as the extinction coefficients, estimated using Expasy software (*web.expasy.org/protparam*) for Alr0088, Alr7579 and All4779 proteins respectively.

**Table 3:** Bacterial strains used in this study.

Bacterial Strains	Description	Source/Reference
*E. coli* strains		
DH5α	F^−^ *recA*41 *endA*1 *gyrA*96 *thi*-1*hsd*R17 (rk^−^mk^−^) *supE*44 *relA* λ Δ*lacU*169	Lab Collection
HB101	F^−^mc^r^ Bm^r^ rhsdS20(r_B_^−^m_B_^−^) *recA*13 *leuB*6 ara-14 proA2 lacY1 galK2 xyl-5 mtl-1 rps (Sm^R^) *gln*V44 λ^−^	Lab Collection
BL21(pLysS)	Cm^r^ F^−^ *ompT* hSdSB (r_B_^−^m_B_^−^) *gal dcm* pLysS (pLysS) (DE3)	Novagen
BL21(pLysS)(pET*alr0088*)	Cm^r^, Cb^r^, *E. coli* BL-21 cells harbouring the plasmid, pET*alr0088*	This study
BL21(pLysS)(pET*alr7579*)	Cm^r^, Cb^r^, *E. coli* BL-21 cells harbouring the plasmid, pET*alr7579*	This study
BL21(pLysS)(pET*all4779*)	Cm^r^, Cb^r^, *E. coli* BL-21 cells harbouring the plasmid, pET*all4779*	This study
Ec(pAM*all4779*)	Kan^r^, HB101 harbouring pAM*all4779* plasmid	This study
HB101 (pRL623+ pRL443)	Donor strain carrying pRL623 (encoding methylase) and conjugal plasmid pRL443	(Wolk, C.P.)
*Anabaena* strains		
*Anabaena* 7120	Wild type strain	Lab Collection
AnpAM	Nm^r^, *Anabaena* 7120 harbouring the plasmid, pAM1956	[26]
An*all4779* ^+^	Nm^r^, *Anabaena* 7120 harbouring the plasmid, pAM*all4779*	This study

The purified recombinant native proteins were individually cross-linked using glutaraldehyde, as described earlier [15], followed by precipitation of protein with cold acetone. The pellet was air dried, solubilised in 1X Laemmli’s buffer by heating at 80°C for 10 min, separated by SDS-PAGE and visualized by staining with CBB G-250.

Molecular mass determination of native purified proteins was carried out by gel filtration chromatography using Superdex HR200 column. The coumn was equilibrated with Tris-NaCl buffer and standard graph obtained using the following proteins: Myosin (200 kDa), β-galactosidase (116 kDa), Phosphorylase-b (97.4 kDa), Bovine Serum Albumin (66 kDa), Chicken Albumin (45 kDa), Carbonic Anhydrase (29 kDa) and RNase A (13.7 kDa). Molecular mass of the three *Anabaena* proteins was calculated from the standard graph on the basis of the elution volume. Presence of protein in different eluates/fractions was detected by measuring absorbance at 280 nm.

### Electrophoretic Mobility Shift Assay (EMSA)

A 75-mer oligonucleotide (10 ng) was end-labelled with γ-^32^P-ATP using Polynucleotide Kinase. The labelled oligo was incubated in the presence of specified concentrations of the Alr0088, Alr7579 and All4779 proteins in binding buffer [20 mM Tris-HCl, pH 8, 1 mM MgCl_2_, 100 mM KCl, 8 mM Dithiothreitol (DTT), 4% sucrose, 80 μg mL^−1^ Bovine Serum Albumin (BSA)] for 30 min at room temperature and electrophoretically separated subsequently on 6% non-denaturing polyacrylamide gel at 150 V for 2 h in 1X Tris-borate EDTA (TBE) buffer. Imaging of the radioactive gel was carried out using Phosphorimager Typhoon Trio Variable mode imager (Wipro-GE-HealthCare, USA).

### Fluorescence Measurements

All three proteins showed maximum excitation at 282 nm. The emission maxima were found to be 310, 340 and 335 nm respectively for Alr0088, Alr7579 and All4779 proteins in 20 mM Tris-HCl, pH 8, 1 mM EDTA buffer. The change in the intensity of the emitted fluorescence was measured in the presence of increasing concentration of ssDNA [poly(dT) or M13 ssDNA]. The graph of relative fluorescence (%) as a function of poly(dT) concentration was used to determine the binding constant for the individual proteins to ssDNA as described earlier [16]. The binding constants were calculated as the reciprocal of the concentration of poly(dT) at which 50% fluorescence compared to the initial 100% was detected. The graph depicting quenching expressed as the ratio of difference in fluorescence to initial fluorescence (ΔF/F_i_) as a function of the ratio of concentrations of poly(dT) and protein was used to determine the binding modes or occlusion site of the proteins as described earlier [17]. It corresponded to the [nt]_poly(dT)_/[Protein] value at the point of saturation of quenching of fluorescence. During titration, solutions were added from concentrated samples and correction for dilution was made as required. All fluorescence measurements were performed with Jasco spectrofluorimeter FP6500 (Japan) using a quartz cuvette of 1 cm path length at room temperature.

### Western Blotting and Immuno-detection

The purified All4779 protein was used to raise specific polyclonal antibody (anti-All4779 antibody) in rabbit. Proteins were extracted from three-day-old wild type and recombinant *Anabaena* cultures in Laemmli’s buffer, separated by 14% SDS-PAGE followed by electroblotting on to nitrocellulose membrane. Immunodetection was carried out using the 1∶5000 dilution of anti-All4779 antibody, followed by secondary anti-rabbit IgG antibody, coupled to alkaline phosphatase and colour development using NBT-BCIP.

### Generation of Recombinant *Anabaena* Strains

The 0.55 kb *Nde*I-*Bam*HI fragment from pET*all4779* was ligated to pFPN vector (Table 2) [18] at the same restriction sites, resulting in the plasmid construct, pFPN*all4779*. The 0.81 kb *Sal*I–*Xma*I fragment from pFPN*all4779* was ligated to pAM1956 vector (Table 2) [19] digested with the identical restriction enzymes. The resulting construct was designated as pAM*all4779* (Table 2). In this construct, the *gfpmut2* gene (coding for Green Fluorescent Protein, GFP) is co-transcribed with the upstream *all4779* gene from the P*_psbA1_* promoter, but the two transcripts are translated independently as described earlier [20]. Recombinant *Anabaena* strain overexpressing All4779 protein (An*all4779^+^*) (Table 3) was generated by introducing the plasmid pAM*all4779* into *Anabaena* by conjugation as described earlier [20], and repeated selection on BG-11 agar plates containing 17 mM NaNO_3_ (BG-11, N^+^) and Neo_25_, till completely segregated cells, uniformly expressing GFP, were obtained.

## Results and Discussion

### Bio-informatic Analysis of Alr0088, Alr7579 and All4779 Proteins

**Figure 1:**
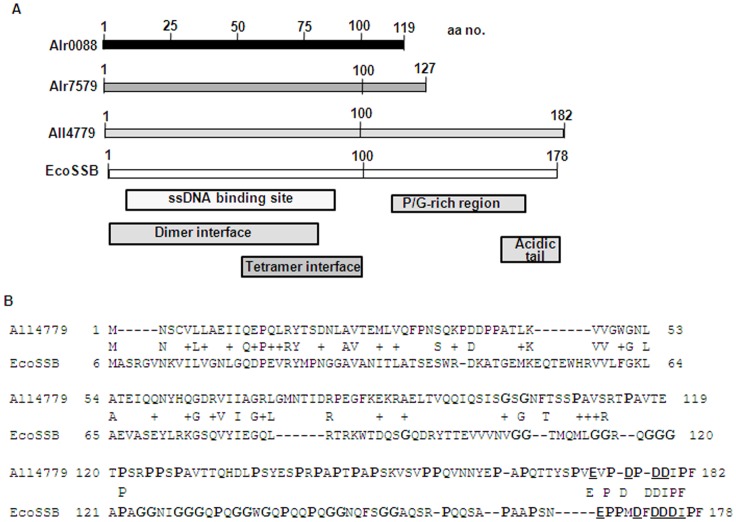
Bioinformatic analysis of Alr0088, Alr7579 and All4779 proteins of *Anabaena* 7120. (A) Conserved Domain Database (CDD) analysis of *Anabaena* Alr0088, Alr7579 and All4779 proteins and *E. coli* SSB (EcoSSB) protein. The OB-fold corresponding ssDNA binding region, dimer and tetramer interfaces for all the proteins are indicated. (B) Comparison of homology between predicted amino acid sequence of All4779 and EcoSSB. The identical amino acids are indicated by letters and similar amino acids with a ‘+’ sign. The proline (P) and glycine (G) residues beyond the OB fold are shown in larger font, while the acidic residues at the C-terminal end are in bold and underlined. The numbers on the left and right hand side correspond to amino acid residues.

The *alr0088*, *alr7579* and *all4779* genes respectively encode 119, 127 and 182 amino acid long polypeptides (Figure 1A) with an estimated molecular mass of 13, 14 and 20 kDa. The prokaryotic SSBs are generally about 160–180 amino acids long, having a molecular mass of 17–18 kDa, except for SsbB of naturally transformable bacteria, which in case of BsSsbB is 113 amino acids long [11]. The SsbA protein of *B. subtilis* is 172 amino acids long, similar in size to EcoSSB [10]. Among the naturally non-transformable bacteria, the smallest known bacterial SSB are from the thermophilic bacteria, *Thermotoga maritima* (TmaSSB) and *T. neapolitana* (TneSSB) consisting of 141 and 142 amino acids respectively, having a single OB-fold domain and a C-terminal domain with the conserved DEPPF terminal amino acids [21]. Among eukaryotes, the *Hs*mtSSB is 133 amino acids long and does not have the region corresponding to 1/3^rd^ of the C-terminal region of EcoSSB [22]. Amino acid sequence analysis using Conserved Domain Database (CDD) [9] revealed the presence of a putative ssDNA-binding OB-fold domain and dimer/tetramer interface within N-terminal half in all three proteins similar to that in *E. coli* SSB (Figure 1A). All4779 additionally had a long C-terminal region similar to that observed for EcoSSB which comprised of a proline-rich region (19 residues) with two glycine residues, as compared to EcoSSB which is glycine rich (21 residues) and has 8 prolines in the corresponding region (Figure 1B). While multiple glycine residues allow flexibility in structure, multiple proline residues provide rigidity and kinks in the structure and thus no ordered structure results in gly-rich or pro-rich regions [23]. The proline-rich region of All4779 would also separate the positively charged N-terminal and the negatively charged C-terminal regions, similar to that in EcoSSB [5]. The N-terminal region of All4779 exhibited 26% identical and 48% similar amino acid residues and a nearly identical acidic tail compared to EcoSSB (Figure 1B). In spite of having an N-terminal OB-fold, P/G rich region and a C-terminal acidic tail, the low homology of All4779 to other known bacterial SSB proteins may possibly account for it not being annotated earlier as SSB-like protein in the genome database of *Anabaena* 7120, unlike Alr0088 and Alr7579 which show a greater homology than All4779 in the OB-fold region.

### Biochemical Characterisation of *Anabaena* Alr0088, Alr7579 and All4779 Proteins

**Figure 2:**
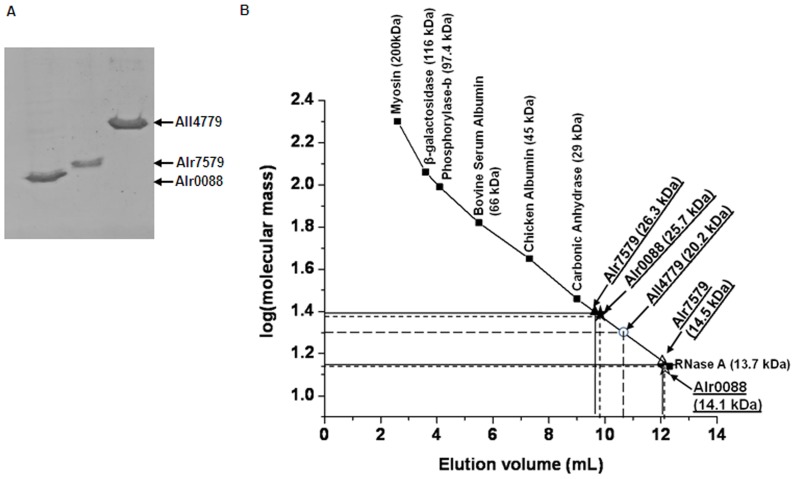
Molecular mass determination of purified native *Anabaena* proteins. (A) Ni-NTA affinity chromatography purified Alr0088, Alr7579 and All4779 proteins separated on 12% SDS-polyacrylamide gel followed by staining with Coomassie Brilliant Blue (CBB) G-250. The purified proteins are indicated by arrows. (B) Elution profile of purified native Alr0088, Alr7579 and All4779 proteins in gel filtration chromatography using Superdex HR200 matrix. A standard graph using the following standard proteins: [Myosin (200 kDa), β-galactosidase (116 kDa), Phosphorylase-b (97.4 kDa), Bovine Serum Albumin (66 kDa), Chicken Albumin (45 kDa), Carbonic Anhydrase (29 kDa) and RNaseA (13.7 kDa)] was drawn to calculate the molecular mass of the eluted native *Anabaena* proteins depending on their elution volume. The position of the eluted proteins has been depicted by ‘star’ and ‘triangle’ symbols. The vertical and horizontal lines from the two symbols indicate the elution volume and the corresponding log of molecular mass.

**Figure 3:**
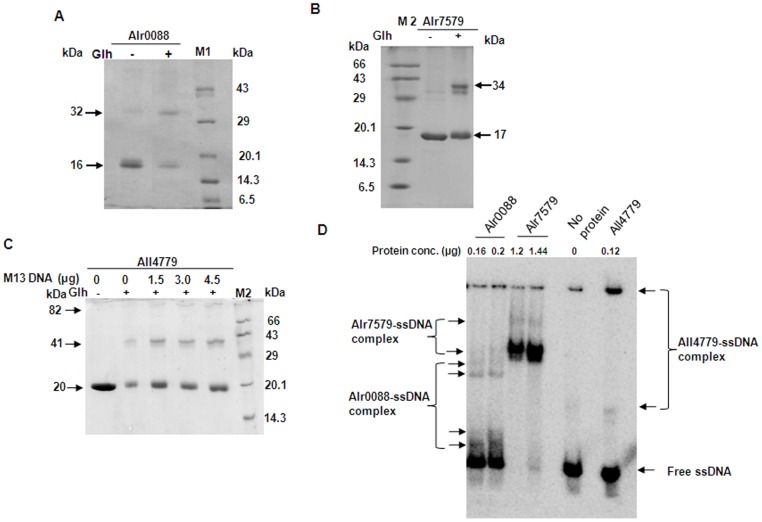
Glutarldehyde (Glh)-aided crosslinking of native purified *Anabaena* SSB-like proteins and their binding to ssDNA. The purified native *Anabaena* proteins (A) Alr0088, (B) Alr7579 and (C) All4779 were cross-linked with Glh in the presence or absence of M13 ssDNA as indicated. The proteins were electrophoretically separated on 12% SDS-polyacrylamide gel followed by staining with Coomassie Brilliant Blue (CBB) G-250. The molecular mass of the protein markers used (M1 and M2) are written to the immediate (right/left) of the marker lane. Different molecular forms of the native *Anabaena* proteins are indicated by the arrows. (D) Electrophoretic Mobility Shift Assay (EMSA) of a γ-^32^P-ATP labeled 75-mer oligonucleotide in the presence of different concentrations of Alr0088, Alr7579 and All4779 proteins. Following *in solution* interaction, the assay mix was separated by 6% non-denaturing PAGE in 1X TBE and radioactive gel imaged using a phosphorimager. The free ssDNA substrate and the different ssDNA-protein complexes formed are indicated.

The *Anabaena* Alr0088, Alr7579 and All4779 proteins overexpressed in *E. coli* BL21(pLysS) cells were purified to near homogeneity using Ni-NTA affinity chromatography (Figure 2A). Presence of dimer and tetramer interfaces in the amino acid sequence (Figure 1A) suggested possibility of formation of multimers by the protein. Alr0088 and Alr7579 were eluted in two distinct fractions and All4779 in a single fraction (Figure 2B) upon separation by gel filtration chromatography using Superdex HR200. On the basis of elution profile of standard proteins on the same matrix, the molecular mass of the different fractions was predicted as 14.1 kDa and 25.7 kDa for Alr0088, 14.5 kDa and 26.3 kDa for Alr7579 and 20.2 kDa for All4779 (Figure 2B). This indicated dimerisation of Alr0088 and Alr7579 proteins as against only the monomeric form detected for All4779 protein. Higher molecular forms of these proteins were not detected even at higher protein concentrations (data not shown). This did not conform to the bioinformatic prediction for the three proteins which indicate the presence of dimeric and tetrameric interfaces (Figure 1A). Further probing of multimeric status was carried out by cross-linking the native proteins with glutaraldehyde followed by separation by SDS-PAGE. Upon cross-linking, the dimeric forms corresponding to 32 kDa for Alr0088 (Figure 3A), 34 kDa for Alr7579 (Figure 3B) and 41 kDa for All4779 (Figure 3C) were detected, with the levels of the dimeric form being lowest for All4779. This could be the reason for the inability to detect a higher molecular weight peak during gel-filtration chromatography for All4779 (Figure 2B). In the presence of M13 ssDNA the levels of the dimeric 41 kDa form as well as a probable tetrameric form of ∼82 kDa increased (Figure 3C). This suggested that All4779 attains the native multimeric conformation preferably in the presence of ssDNA. In case of Alr0088 and Alr7579, no effect on the levels of the dimeric form or generation of tetrameric form was observed even with ssDNA (data not shown). In general, bacterial SSB proteins function as tetramers [1], with the exception of thermophilic group of organisms (Thermus spp.) and the radioresistant microbe *Deinococcus radiodurans* [24] which function as dimer. However, the protomers of SSB of these organisms are twice the size of *E. coli* SSB and contain two OB-folds per monomer [24].

The DNA binding ability of the SSB-like proteins was assessed by Elctrophoretic Mobility Shift Assay (EMSA) and fluorescence quenching techniques. Multiple shifts in the mobility of the 75-mer ss oligonucleotide was observed in the presence of Alr0088 (Fig. 3D). Increase in concentration beyond 0.2 μg Alr0088 did not result in any further shifts in mobility (data not shown). Alr7579 decreased the mobility of the 75-mer oligo only when used at very high concentrations of 1.2–1.4 μg (Fig. 3D). Presence of Alr7579 also resulted in detection of multiple bands differing in their mobility, but majority of the complex formed, even when low concentrations of 0.12 μg of All4779 was used, was detected near the well (Fig. 3D). Based on this, the binding efficiency for ssDNA seems to be maximum for All4779, followed by Alr0088 and the least for Alr7579. The binding affinity for each of these proteins for ssDNA was calculated by fluorescence quenching technique using poly(dT) as the ssDNA substrate.

**Figure 4:**
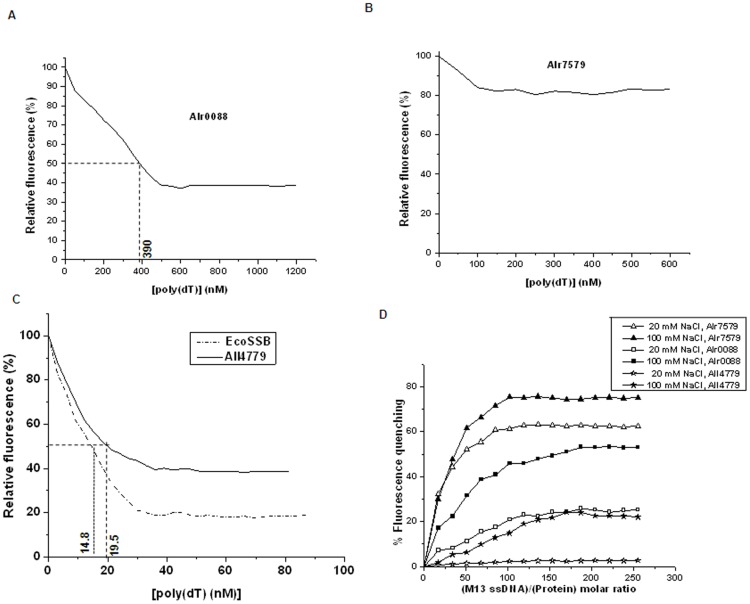
Relative quenching of fluorescence of native purified *Anabaena* SSB-like proteins and EcoSSB as a function of ssDNA concentration. (A–C) Quenching of fluorescence in 20 mM NaCl as a function of poly(dT) concentration of (A) Alr0088, (B) Alr7579 and (C) All4779 and purified EcoSSB (commercially available, Sigma) proteins represented as relative fluorescence, considering the observed fluorescence in the absence of any poly(dT) as 100%. The horizontal line designates the point on the graph wherein relative fluorescence is 50% and the corresponding vertical line indicates the concentration of poly(dT) at which it is achieved. Reciprocal of this concentration corresponds to the binding constant of the protein for poly(dT). (D) Percent fluorescence quenching of Alr0088, Alr7579 and All4779 proteins as a function of molar ratio of M13ssDNA and protein in the presence of 20 mM or 100 mM NaCl. The fluorescence quenching in the absence of M13ssDNA is considered as 0%.

The relative fluorescence of native *Anabaena* proteins Alr0088, Alr7579 and All4779, was measured as a function of increasing concentration of poly(dT) at 20 mM NaCl. The relative fluorescence of (i) Alr0088 decreased to a maximum of 40% with ∼450 nM poly(dT) (Figure 4A), (ii) Alr7579 showed less than 20% decrease (Figure 4B), and (iii) All4779 up to 40% of the initial fluorescence, but at much lower concentrations (∼35 nM) of poly(dT) (Figure 4C). Based on this, the binding constant, as an average of three independent experiments, was calculated as 2.56±0.4×10^6^ M^−1^ for Alr0088, 5.13±0.71×10^7^ M^−1^ for All4779 and 6.76×10^7^ M^−1^ for EcoSSB (Figure 4C), which was comparable to that reported for EcoSSB (5.5±1.5×10^7^ M^−1^) [4]. In the absence of C-terminal acidic tail, the binding affinity for ssDNA has been shown to increase 10-fold in case of EcoSSB [4], as well as for HsmtSSB, which lacks the C-terminal tail, calculated as 4×10^8^ M^−1^ [25]. However, the reverse was found to be true in case of *Anabaena* 7120, with Alr0088 which lacks the acidic tail, having 10-fold lower binding affinity than All4779. This could be due to the additional absence of the P/G-rich region as well in Alr0088.

The inability of Alr7579 to bind poly(dT) raised questions on whether the OB-fold, responsible for binding ssDNA [1] is active in Alr7579. To test this, a larger ssDNA, such as M13 ssDNA was used as a substrate. A 60–70% quenching of the fluorescence of Alr7579 was observed with the 7 kb M13 ssDNA, the efficiency being higher at high NaCl concentration (Figure 4D), which allows formation of a more compact structure of ssDNA. The quenching of fluorescence of Alr7579 was not observed with thermally denatured M13 ssDNA (data not shown). M13 ssDNA is known to form secondary structures [26], which are disrupted at higher temperature. This suggested that Alr7579 may be recognising secondary structures formed with long ssDNA, rather than short stretches of linear ssDNA. Both Alr0088 and All4779 also bound M13 ssDNA at high salt concentration, but with lower efficiency, the quenching of fluorescence being 40% and 18% respectively (Figure 4D). While low quenching of fluorescence of Alr0088 by M13 ssDNA was observed at low NaCl (Figure 4D), indicating low level interactions, no such interaction was observed for All4779 (Figure 4D).

**Figure 5:**
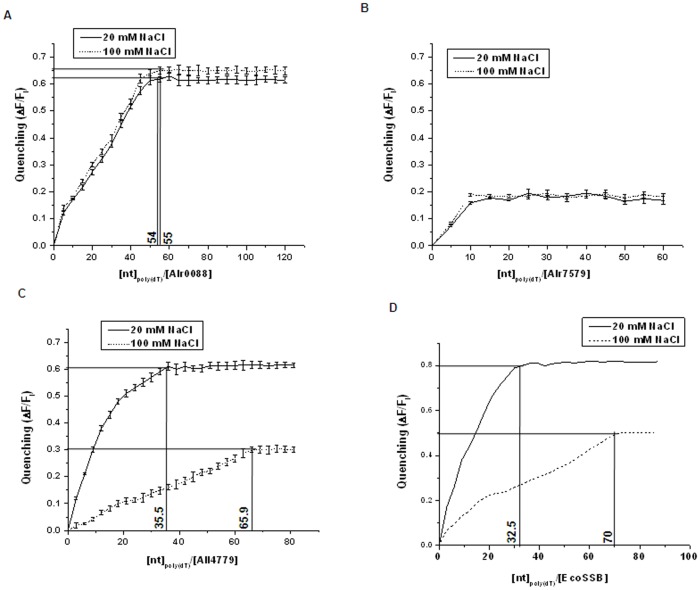
Quenching of fluorescence of native purified *Anabaena* SSB-like proteins compared with that of EcoSSB. The quenching of fluorescence of (A) Alr0088, (B) Alr7579, (C) All4779 and (D) EcoSSB in the presence of 20 mM or 100 mM NaCl was expressed as a ratio of change in fluorescence (ΔF) and initial fluorescence (F_i_). The (ΔF/F_i_) was expressed as a function of ratio of concentrations of poly(dT) and protein. The horizontal lines indicate the point of saturation and the vertical lines drawn from the point of saturation indicate the probable length of ssDNA bound by one molecular unit of the protein.

In general, SSB proteins interact with ssDNA in multiple binding modes, differing in the number of OB-folds which interact with the ssDNA. In the (SSB)_35_ mode, approximately 35 nucleotides of ssDNA interact with two subunits of the Ssb tetramer, while in (SSB)_65_ mode, ∼65 nucleotides of ssDNA wrap around all four subunits, which is more favoured at higher salt concentrations [25]. Based on the quenching of fluorescence (ΔF/F_i_) of the three *Anabaena* proteins with poly(dT) at low (20 mM) NaCl and high (100 mM) NaCl concentrations, binding modes or occlusion size for each of the protein determined. A single binding mode of 54–55 nucleotides was estimated for Alr0088, which was independent of NaCl concentration (Figure 5A). No significant quenching of fluorescence of Alr7579 was observed at low or high concentrations of NaCl (Figure 5B), while two binding modes dependent on NaCl concentration was observed for All4779 (Figure 5C). The binding size was found to be 35.5 nucleotides at 20 mM NaCl and 65.9 nucleotides at 100 mM NaCl for All4779 (Figure 5C), and 32.5 and 70 nucleotides at 20 mM and 100 mM NaCl respectively for EcoSSB under identical experimental conditions, comparable to the (SSB)_35_ and (SSB)_65_ modes of binding, at low and high salt concentrations respectively, shown for EcoSSB [1], [27]. Since, (SSB)_65_ mode of binding requires the binding of ssDNA to the tetrameric form of SSB [27], and molecular form corresponding to a tetramer of All4779 was very low, the quenching of fluorescence of All4779 at higher NaCl was lower than that at lower NaCl (Figure 5C), as well as that observed with EcoSSB (Figure 5D).

Thus, though all the three proteins i.e. Alr0088, Alr7579 and All4779 bind ssDNA, their binding affinity and modes of binding are distinct and among these, the binding ability as well as binding modes of All4779 was quite similar to other known bacterial SSBs. The presence of (P/G)-rich spacer and a near identical C-terminal acidic tail, suggests that in *Anabaena* 7120, All4779 may also be performing *in vivo* functions similar to those carried out by the typical bacterial SSB proteins. Since, overexpression of bacterial SSBs are known to influence the repair of stress induced DNA damage [28], [29], thereby enhancing tolerance to DNA damaging stresses, a similar role for All4779 was assessed in *Anabaena* 7120.

### Physiological Role of All4779 Protein in *Anabaena* 7120

**Figure 6:**
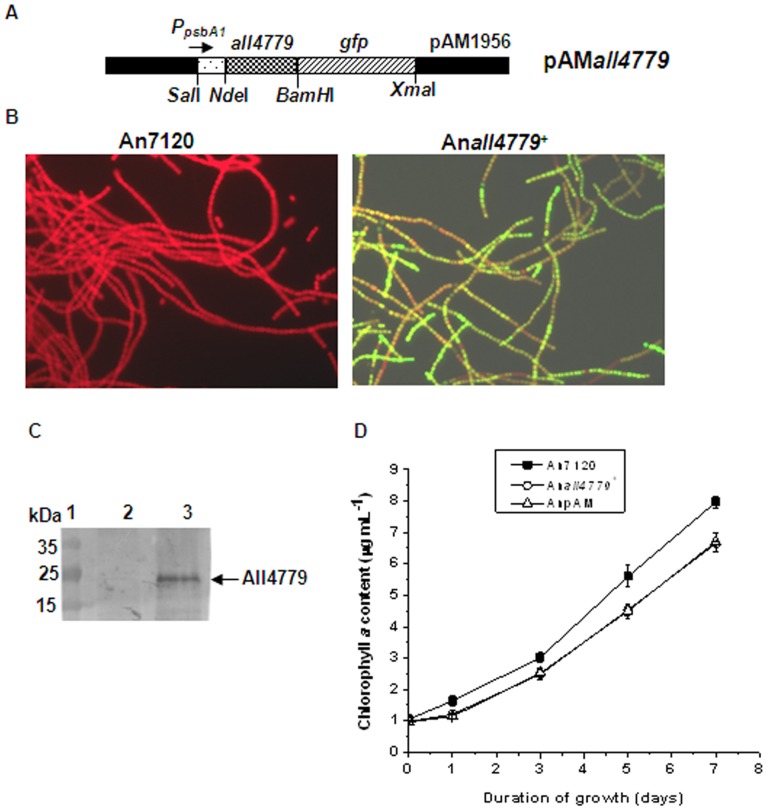
Construction of recombinant *Anabaena* strain overexpressing All4779 protein. (A) Schematic diagram of the plasmid construct, pAM*all4779* used for overexpression of All4779 protein in *Anabaena*. The different restriction enzymes used for cloning are indicated. (B) Fluorescence microphotograph (600X magnification) [using Hg-Arc lamp (excitation 470 nm, emission 508 nm)] of *Anabaena* 7120 [An7120] and recombinant strain, An*all4779* ^+^, grown for 3 days in BG-11, N^−^ media. (C) Protein extracts from *Anabaena* 7120 (lane 1) and An*all4779* ^+^ (lane 2) were separated by 12% SDS-PAGE, followed by blotting on to nitrocellulose membrane and immunodetection of All4779 protein using anti-All4779 antibody. The cross-reacting All4779 protein is indicated by an arrow. Equal loading controls are shown below the blot. Other details were as described in legend to Figure 2. (D) Growth profile of wild type *Anabaena* 7120 and recombinant *Anabaena* strain, An*all4779* ^+^ and AnpAM under nitrogen-fixing conditions over a period of 7 days. Growth was measured in terms of increase in chlorophyll *a* content. Recombinant strains were grown in presence of neomycin while wild type was grown without antibiotic.

The All4779 protein was overexpressed *in trans* from the plasmid pAM*all4779* (Table 2, Figure 6A) in the recombinant *Anabaena* strain, An*all4779* ^+^ (Table 3). Due to growth under continuous illumination, the expression of the All4779 protein from the light-inducible *psb*A1 promoter was expected to be constitutive. Co-overexpression of the Green Fluorescent Protein (GFP), coded by *gfpmut2* in the pAM*all4779* plasmid, provided a handy tool to distinguish the fully segregated recombinant An*all4779^+^* strain exhibiting green fluorescence, from the wild type *Anabaena* 7120 which exhibited red fluorescence upon excitation with λ_470_ light (Figure 6B). It also ensured expression of the upstream gene. The overexpression of All4779 in An*all4779* ^+^ cells was indeed confirmed by immunodetection with anti-All4779 antibody (Figure 6C). Under normal growth conditions, the nitrogen-fixing cultures of An*all4779* ^+^ grew marginally slower than the wild type *Anabaena* 7120 cultures, and at rates comparable to the recombinant *Anabaena* strain harbouring pAM1956 vector, AnpAM (Table 3) [30] (Figure 5D). This is possibly due to the presence of neomycin in the growth medium used for recombinant strain.

**Figure 7:**
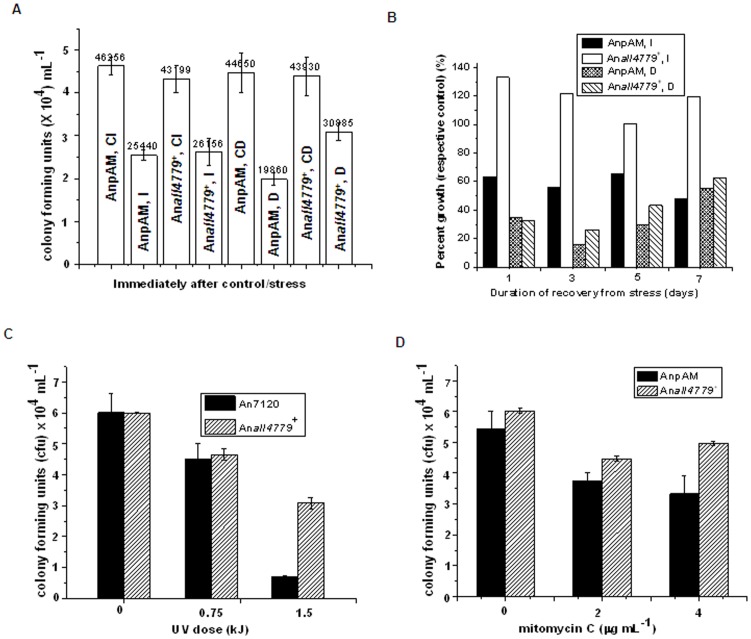
Effect of All4779 overexpression on the survival and tolerance of *Anabaena* to DNA-damage inducing stresses. (A and B) Three day-old cultures were concentrated to 10 μg chl*a* density mL^−1^ and exposed to 6 kGy of ^6^°Co γ-irradiation or to 6 days of desiccation. (A) Survival was measured in terms of colony forming units immediately after irradiation (I) or desiccation (D) and compared with the respective unirradiated control (CI) or undesiccated control (CD). (B) The stressed and control cultures were washed, inoculated in fresh BG-11, N^−^, Neo_12.5_ and allowed to recover under normal growth conditions for 7 days. Growth during post-irradiation/desiccation recovery was measured in terms of chlorophyll *a* content and expressed as percent of respective unirradiated/undesiccated controls. (C and D) Three-day-old cultures of recombinant strains AnpAM and An*all4779* ^+^ were concentrated to 10 μg chl*a* mL^−1^ density. (C) An 100 μl aliquot was spread on the corresponding BG-11, N^−^, Neo_25_ agar plates and exposed to UV-B (0–1.5 kJ) (D) Culture aliquots were exposed to mitomycinC (0–4 μg ml^−1^) for 30 min in liquid media followed by plating 100 μl on BG-11, N^−^ Neo_25_ agar plate. Colonies were counted after 10 days of incubation at 27°±2°C with constant illumination.

The effect of overexpression of All4779 on the ability of *Anabaena* 7120 to tolerate DNA damage inducing stresses was analysed in response to two distinct types of DNA damages i.e. (i) γ-irradiation and desiccation which cause single strand and double strand breaks, and (ii) UV-B and mitomycinC which cause formation of pyrimidine dimers and DNA adducts respectively. The empty vector control recombinant strain, AnpAM exhibited about 55% and 44% survival upon exposure to 6 kGy of ^6^°Co γ-rays or 6 days of desiccation respectively (Figure 7A). Upon constitutive overexpression of All4779 in An*all4779* ^+^ cells, the survival increased to about 60% after exposure to 6 kGy of γ-rays and 70% after 6 days of desiccation (Figure 7A). The recovery of irradiated cultures of *Anabaena*, measured in terms of chlorophyll *a* content increased from about 50% to over 100% in cells overexpressing All4779 (Figure 7B) suggesting better tolerance to radiation. Such correlation was however, not found in post desiccation recovery, (Figure 7B), possibly due to additional stresses, such as osmotic stress experienced during desiccation followed by rehydration of these cells. Of the other two SSB-like proteins of *Anabaena*, overexpression of Alr0088 decreased the radiation tolerance of *Anabaena*, while that of Alr7579 had no effect [14]. This suggested that All4779 is the typical bacterial SSB of *Anabaena*, involved in the repair of single and double strand breaks in DNA, possibly as part of a larger DNA repair complex, which is yet to be identified.

Overexpression of All4779 was also beneficial in protection against stresses which caused formation of DNA adducts. The survival of AnpAM was about 75% and 11.8% respectively upon exposure to 0.75 and 1.5 kJ m^−2^ of UV-B irradiation, which increased to 77% and 50% respectively in An*all4779* ^+^ cells, overexpressing All4779 protein (Figure 7C). The beneficial effect of the constitutive overexpression of All4779 was more pronounced when exposed to higher doses (1.5 kJ m^−2^) of UV-B (Figure 7C), while at lower dose of (0.75 kJ m^−2^), that of Alr0088 was more benficial [14]. AnpAM cells exhibited 50% survival upon exposure to 4 μg mitomycinC mL^−1^ for 30 min, which increased to 85% upon overexpression of All4779 in An*all4779* ^+^ cells (Figure 7D), comparable to that observed upon overexpression of Alr7579, but lower than that with Alr0088 [14]. Thus, the presence of high levels of All4779 in *Anabaena* possibly decreased the net damage to DNA, both in terms of single and double stranded breaks as well as formation of DNA adducts, possibly by efficient repair of the damaged DNA. Overexpression of SSB has been shown to be beneficial by aiding DNA repair in *E. coli* cells [28].

Thus, All4779 is the major typical bacterial SSB of *Anabaena* 7120 in terms of structural domains, binding to ssDNA and physiological role in DNA repair. The genes coding for the two atypical truncated annotated SSB proteins, Alr0088 and Alr7579 may have arisen due to gene duplication as suggested for PriB, a dimeric protein with only OB-fold and capable of binding ssDNA [31] and may be involved in other functions such as replication and recombination. The unicellular cyanobacterium, *Synechocystis* PCC6803 has been shown to be naturally transformable with possible involvement of competence proteins, ComA (Slr0197) [32] and ComF (Slr0388) [33]. The orthologs of these genes are also found in *Anabaena* 7120, annotated as *all3087* and *alr2926* respectively (http://genome.microbedb.jp/cyanobase/Anabaena), suggesting the possibility of *Anabaena* being also naturally transformable, though this needs to be ascertained. Thus, as has been observed in case of the naturally transformable *B. Subtilis*, the naturally C-terminal truncated BsSsbB, is involved in competence by protecting the incoming DNA [10], [11], a similar role may also be associated with Alr0088 or/and Alr7579, both of which bear moderate homology to BsSsb, though this needs to be ascertained. The acidic tail, characteristic of most SSBs, has been shown to be the site of interaction with DNA repair proteins for *E. coli* SSB [3]. Owing to the presence of an acidic tail, All4779 upon overexpression offers better protection from DNA-damage when subjected to different DNA-damage-inducing stresses. Based on data presented, we propose that All4779 be re-annotated as the gene coding for typical single stranded DNA binding protein (SSB) and the corresponding ORF be annotated as the *ssb* gene of *Anabaena* 7120.

## References

[1] Lohman TM, Ferrari ME (1994) *Escherichia coli* single-stranded DNA-binding protein: multiple DNA-binding modes and cooperativities. Annu. Rev. Biochem. 63: 527–570. 10.1146/annurev.bi.63.070194.002523 7979247

[2] Carlini L, Curth U, Kindler B, Urbanke C, Porter RD (1998) Identification of amino acids stabilizing the tetramerization of the single stranded DNA binding protein from *Escherichia coli*. FEBS Lett. 430: 197–200. 10.1016/s0014-5793(98)00655-3 9688537

[3] Shereda RD, Kozlov AG, Lohman TM, Cox MM, Keck JL (2008) SSB as an organizer/mobilizer of genome maintenance complexes. Crit. Rev. Biochem. Mol. Biol. 43: 289–318. 10.1080/10409230802341296 PMC2583361 18937104

[4] Kozlov AG, Cox MM, Lohman TM (2010) Regulation of single-stranded DNA binding by the C termini of *Escherichia coli* single-stranded DNA binding (SSB) protein. J. Biol. Chem. 285: 17246–17252. 10.1074/jbc.M110.118273 PMC2878057 20360609

[5] Eggington JM, Haruta N, Wood EA, Cox MM (2004) The single-stranded DNA- binding protein of *Deinococcus radiodurans* . BMC Microbiol. 4: 2 doi 10.1186/1471-2180-4-2 10.1186/1471-2180-4-2 PMC331404 14718065

[6] Apte SK (2001) Coping with salinity/water stress: Cyanobacteria show the way. Proc. Indian Natl. Acad. Sci (PINSA) B67: 285–310.

[7] Singh H, Fernandes T, Apte SK (2010) Unusual radioresistance of nitrogen-fixing cultures of *Anabaena* strains. J. Biosci. 35: 427–434. 10.1007/s12038-010-0048-9 20826952

[8] Singh H, Anurag K, Apte SK (2013) High radiation and desiccation tolerance of nitrogen-fixing cultures of the cyanobacterium *Anabaena* sp. strain PCC7120 emanates from genome/proteome repair capabilities. Photosynth. Res. 118: 71–81. 10.1007/s11120-013-9936-9 24122300

[9] Marchler-Bauer A, Lu S, Anderson JB, Chitsaz F, Derbyshire MK, et al (2011) CDD: a Conserved Domain Database for the functional annotation of proteins. Nucleic Acids Res. 39: 225–229. 10.1093/nar/gkq1189 PMC3013737 21109532

[10] Yadav T, Carrasco B, Myers AR, George NP, Keck JL, et al (2012) Genetic recombination in *Bacillus subtilis*: a division of labor between two single-strand DNA-binding proteins. Nuc. Acid Res. 40: 5546–5559. 10.1093/nar/gks173 PMC3384303 22373918

[11] Kidane D, Ayora S, Sweasy JB, Graumann PL, Alonso JC (2012) The cell pole: the site of cross talk between the DNA uptake and genetic recombination machinery. Crit. Rev. Biochem. Mol. Biol. 47: 531–555. 10.3109/10409238.2012.729562 PMC3490228 23046409

[12] Castenholz RW (1988) Culturing methods for cyanobacteria. Methods Enzymol. 167: 68–93.

[13] Mackinney G (1941) Absorption of light by chlorophyll solutions. J. Biol. Chem. 140: 315–322.

[14] Kirti A, Rajaram H, Apte SK (2013) Characterization of two naturally truncated, Ssb-like proteins from the nitrogen-fixing cyanobacterium, *Anabaena* sp. PCC7120. Photosynth. Res. 118: 147–154. 10.1007/s11120-013-9904-4 23928723

[15] Wadsworth RI, White MF (2001) Identification and properties of the crenarchaeal single-stranded DNA binding protein from *Sulfolobus solfataricus*. Nucleic Acids Res. 29: 914–920. 10.1093/nar/29.4.914 PMC29618 11160923

[16] Molineux IJ, Pauli A, Gefter ML (1975) Physical studies of the interaction between the *Escherichia coli* DNA binding protein and nucleic acids. Nucleic Acids Res 2: 1821–1837. 1103088 10.1093/nar/2.10.1821 PMC343550

[17] Lohman TM, Overman LB (1985) Two binding modes in *Escherichia coli* single strand binding protein-single stranded DNA complexes. Modulation by NaCl concentration. J Biol. Chem. 260: 3594–3603. 3882711

[18] Chaurasia AK, Parasnis A, Apte SK (2008) An integrative expression vector for strain improvement and environment applications of nitrogen-fixing cyanobacterium *Anabaena* sp. strain PCC7120. J. Microbiol. Methods. 73: 133–141. 10.1016/j.mimet.2008.01.013 18367274

[19] Yoon HS, Golden JW (1998) Heterocyst pattern formation controlled by a diffusible peptide. Science. 282: 935–938. 10.1126/science.282.5390.935 9794762

[20] Raghavan PS, Rajaram H, Apte SK (2011) Nitrogen status dependent oxidative stress tolerance conferred by overexpression of MnSOD and FeSOD proteins in *Anabaena* sp. strain PCC7120. Plant Mol. Biol. 77: 407–417. 10.1007/s11103-011-9821-x 21882041

[21] Olszewski M, Grot A, Wojciechowski M, Nowak M, Mickiewicz M, et al (2010) Charcterization of exceptionally thermostable single-stranded DNA-binding proteins from *Thermotoga maritima* and *Theromotoga neapolitana* . BMC Microbiol. 10: 260 doi.10.1186/1471-2180-10-260 20950419 10.1186/1471-2180-10-260 PMC2964679

[22] Curth U, Urbanke C, Greipel J, Gerberding H, Tiranti V, et al (1994) Single-stranded-DNA binding proteins from human mitochondria and *Escherichia coli* have analogous physicochemical properties. Eur. J. Biochem. 221: 435–443. 10.1111/j.1432-1033.1994.tb18756.x 8168532

[23] Belts MJ, Russell RB (2003) Amino acid properties and consequences of substitutions. In Bioinformatics for Geneticists. Ed. Barnes MR and Gray IC. John Wiley and sons, 289–316.

[24] Bernstein DA, Eggington JM, Killoran MP, Misic AM, Cox MM, et al (2004) Crystal structure of the *Deinococcus radiodurans* single-stranded DNA-binding protein suggests a mechanism for coping with DNA damage. Proc. Natl. Acad. Sci. 101: 8575–8580. 10.1073/pnas.0401331101 PMC423236 15159541

[25] Curth U, Genschel J, Urbanke C, Greipel J (1996) *In vitro* and *in vivo* function of the C-terminus of *Escherichia coli* single-stranded DNA binding protein. Nucleic Acids Res. 24: 2706–2711. 10.1093/nar/24.14.2706 PMC145992 8759000

[26] Reckmann B, Grosse F, Urbanke C, Frank R, Blocker H, et al (1985) Analysis of secondary structures in M13mp8 (+) single-stranded DNA by the pausing of DNA Polymerase α. Eur. J. Biochem. 152: 633–643. 10.1111/j.1432-1033.1985.tb09242.x 2996896

[27] Bujalowski W, Lohman TM (1986) *Escherichia coli* single-strand binding protein forms multiple, distinct complexes with single-stranded DNA. Biochem. 25: 7799–7802. 10.1021/bi00372a003 3542037

[28] Moreau PL (1987) Effects of overproduction of single-stranded DNA-binding protein on RecA protein-dependent processes in *Escherichia coli*. J. Mol. Biol. 194: 621–634. 10.1016/0022-2836(87)90239-7 3309327

[29] Moreau PL (1988) Overproduction of single-stranded-DNA-binding protein specifically inhibits recombination of UV-irradiated bacteriophage DNA in *Escherichia coli*. J. Bacteriol. 170: 2493–2500. 10.1128/jb.170.6.2493-2500.1988 PMC211161 2836358

[30] Rajaram H, Apte SK (2010) Differential regulation of *groESL* operon expression in response to heat and light in *Anabaena*. Arch. Microbiol. 192: 729–738. 10.1007/s00203-010-0601-9 20596696

[31] Ponomarev VA, Makarova KS, Aravind L, Koonin EV (2003) Gene duplication with displacement and rearrangement: origin of the bacterial replication protein PriB from the single-stranded DNA-binding protein, Ssb. J. Mol. Microbiol. Biotechnol. 5: 225–229. 10.1159/000071074 12867746

[32] Yura K, Toh H, Go M (1999) Putative mechanism of natural transformation as deduced form genome data. DNA Res. 6: 75–82. 10.1093/dnares/6.2.75 10382965

[33] Nakasugi K, Svenson CJ, Neilan BA (2006) The competence gene, *comF*, from *Synechocystis* sp. strain PCC6803 is involved in natural transformation, phototactic motility and piliation. Microbiol. 152: 3623–3631. 10.1099/mic.0.29189-0 17159215

